# Medicago Sativa Stems—A Multi-Output Integrated Biorefinery Approach

**DOI:** 10.3390/polym17121709

**Published:** 2025-06-19

**Authors:** Adrian Cătălin Puițel, George Bârjoveanu, Cătălin Dumitrel Balan, Mircea Teodor Nechita

**Affiliations:** Faculty of Chemical Engineering and Environmental Protection “Cristofor Simionescu”, Technical University “Gheorghe Asachi” Iasi, Bd. Prof. Dimitrie Mangeron, No. 73, 700050 Iaşi, Romania; adrian-catalin.puitel@academic.tuiasi.ro (A.C.P.); george.barjoveanu@academic.tuiasi.ro (G.B.); catalin-dumitrel.balan@academic.tuiasi.ro (C.D.B.)

**Keywords:** biorefinery, waste biomass, alfalfa, protein extracts, structural carbohydrates, pulping, papermaking fibers

## Abstract

This study presents an investigation on the potential of using one-year-old field-stored *Medicago sativa* (alfalfa) as a raw material for a multi-output biorefinery. The main objective was to fractionate the biomass into valuable components—crude protein, hemicellulose-derived polysaccharides, lignin, and cellulose—and to explore the latter’s suitability in papermaking. To this end, three pretreatment strategies (water, alkaline buffer, and NaOH solution) were applied, followed by soda pulping under varying severity conditions. Both solid and liquid fractions were collected and chemically characterized using FTIR, HPLC, and standardized chemical methods. Water-based pretreatment was most effective for protein extraction, achieving over 40% protein content in precipitated fractions. The harshest pulping conditions (20% NaOH, 160 °C, 60 min) yielded cellulose-rich pulp with high glucan content, while also facilitating lignin and hemicellulose recovery from black liquor. Furthermore, the pulps derived from alfalfa stems were tested for papermaking. When blended with old corrugated cardboard (OCC), the fibers enhanced tensile and burst strength by 35% and 70%, respectively, compared to OCC alone. These findings support the valorization of unexploited alfalfa deposits and suggest a feasible biorefinery approach for protein, fiber, and polymer recovery, aligned with circular economy principles.

## 1. Introduction

The “historical” roots of *Medicago sativa*, also known as alfalfa or Lucerne (MS), stretch back to the 6th millennium BC, and this plant is considered the oldest fodder with a known name [1]. It took 8000 years for this versatile plant to spread around the world [1], but nowadays, it is recognized as either the “king” [2,3] or “queen” [1,4] of forages, being considered as the most important forage legume [5,6]. It is a perennial plant that can live up to 20 years, but only the first 4–5 years are considered economically productive; in this period, it can be harvested from two to six or seven times per year, depending upon irrigation, fertilizing, and weather conditions [7]. Over time, besides its well-known forage qualities, this adaptable plant revealed some complementary benefits [8] such as (i) weed suppression—MS is an environmentally friendly culture that allows crop producers to diminish herbicide consumption [9,10]; (ii) high ability for biological nitrogen fixation, improving soil quality [11,12]; (iii) sediment and runoff reduction—soil erosion prevention by retaining flow and decreasing soil loss [13,14]; (iv) phytochemical and pharmaceutical properties [15,16]; (v) being a food source for bees and other pollinators [17,18]—approximately 30% of the honey in the U.S.A. comes from alfalfa fields [19]; and (vi) soil phytoremediation of toxic elements [20,21].

In the past decades, MS has made the transition from feed to food [22,23], as an alternative protein source for human consumption [24]. In 2009, the European Food Safety Authority published an “Opinion on the safety of ‘Alfalfa protein concentrate’ as food,” concluding that alfalfa protein concentrate is safe for human consumption as a food supplement (following specific rules of consumption) [25]. In the same year, the Commission of the European Communities promoted a decision that authorized the commercialization of a leaf extract from Lucerne (*Medicago sativa*) as a novel food or novel food ingredient [26]. Nowadays, alfalfa sprouts represent a widespread fresh vegetable dish commonly consumed in salads [27] and as a dietary supplement in the form of powder, capsules, and tablets [28].

Owing to this multitude of practical applications, the surfaces cultivated with MS and, consequently, the MS market are experiencing continuous growth. According to Fortune Business Inside [29], the global MS market was valued at USD 25.66 billion in 2023 and is projected to reach an impressive USD 46.56 billion in 2032, which is almost double in less than a decade.

It is the plant’s chemical composition that makes this vegetable attractive [6]. The high protein content [30], accompanied by vitamins and other compounds, makes MS a highly appreciated feed and, recently, a food source. The protein content is higher in the leaves than in the stem, and it is much easier to extract protein from fresh green leaves than from the dried plant [31,32]. Prior to harvesting, 60% to 80% of total plant nitrogen is in the form of soluble proteins; after cutting, during mechanical processing and storage, 15 to 25% of the soluble proteins are hydrolyzed into small peptides, free amino acids, and amines while the remaining soluble proteins are partly denatured to become insoluble [33]. The insoluble protein fraction remains relatively stable during ensiling, storage, or haying, ensuring the nutritional quality of the forage [33]. Drying and aging in any storage form result in a relatively slow decay of protein content caused by chemical degeneration and mechanical loss of the leaves [34,35,36,37]. The leaf-to-stem ratio is a crucial parameter when it comes to protein recovery yields [12]. It is a well-known fact that all the mechanical operations performed after harvesting with the dried plant generate losses in the leaf content that directly affect the protein concentration and consequently the forage quality [34,35,36,37]. When the stored alfalfa quality declines, producers must invest in additional supplements to compensate for the lack of nutrients in the livestock’s diet.

Overstocking, premature access to fresh feed in early spring, or unexpected dropping of farmed animal populations produce large amounts of unexploited deposits of MS that continuously lose nutritional quality. They still can be used as forage, but the results in terms of meat and/or milk quality and yields are lower and lower over time. Therefore, besides the conventional uses as feed and food, the exploitation of the industrial potential of alfalfa and/or unused stocks of alfalfa for the production of other goods may raise the economic benefits for MS cultivators and farmers. From this point of view, several attempts were made for MS valorization, other than its routine use for food/feed, such as the production of electricity [38], biochar [39], biogas [40], biodegradable plastic polymers [41], adhesive [42], bioethanol [43,44], protein and ethanol [45], cellulosic ethanol and paper pulp [46], powder cellulose [47], paper [48,49], nanoparticles [50], and various polysaccharides [51].

The main goal of this work is to investigate the technical utilization potential of one-year-old field-stored Medicago sativa stems (MSSs) as raw material for a biorefinery aiming to produce crude protein, hemicellulose-derived polysaccharides, cellulose, and lignin. An initial objective of our work was to investigate the chemical composition variability of MS and then to investigate how three preliminary treatment alternatives would impact the protein and polysaccharides yield and types and finally to characterize the resulting chemical components.

## 2. Materials and Methods

### 2.1. Materials

**Figure 1:**
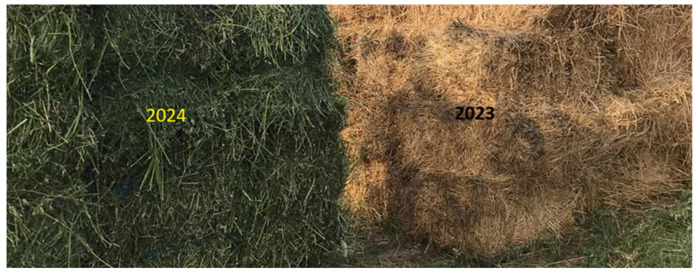
Alfalfa bales stored in the field: fresh (2024), one year old (2023).

The alfalfa samples were donated by local farmers from Drăgușeni Village, Suceava County, Romania. The raw materials for this study were primarily field-dried alfalfa hay that was baled and stored in the field for a year (from July 2023 to July 2024). For comparison, a year later (2024), alfalfa hay from the same harvest (third cut) was gathered and stored in the same way (Figure 1). Additionally, leaf and stem separation and oven drying were applied to green-state (freshly harvested) MS in order to ascertain the percentage of leaves and stems.

All chemicals and reagents used were of analytical purity. Solutions of 99% purity of cellobiose, glucose, xylose, galactose, arabinose, and mannose, provided by Flucka Buchs, Buchs, Switzerland, were used to obtain the calibration curves.

### 2.2. Experimental Approach

**Figure 2:**
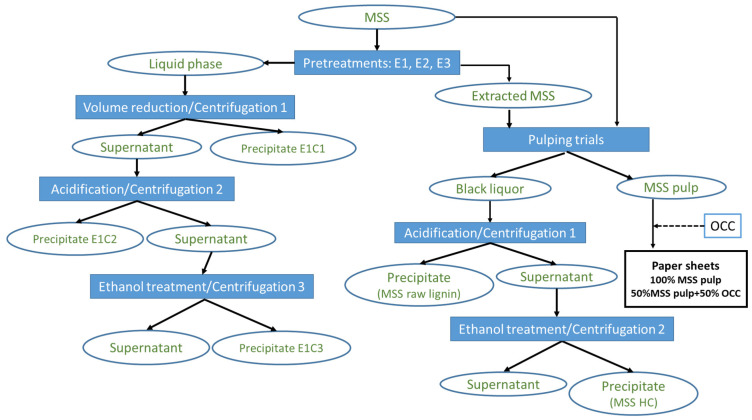
Experimental strategy for Medicago Sativa stem biorefinery, where MSS represents Medicago Sativa stems; E1, E2 and E3 represent the specific pretreatments; E1C1, E1C2, and E1C3 represent the solid phases resulting after the processing of the liquid phase resulting from E1; OCC represents old corrugated cardboard; MSS HC represents the hemicelluloses separated from black liquors.

Figure 2 presents the streamlined experimental methodology for the MS biorefinery. Three distinct pretreatment experiments (E1, E2, and E3) were performed on MSS samples. The liquid and solid phases produced were subjected to further processing. The L_MSS_ was subjected to two successive precipitation stages, while the S_MSS_ went to pulping. Three different experimental conditions (PT1, PT2, and PT3) were used for the pulping experiments. Raw MSS samples were also pulped under PT1, PT2, and PT3 conditions for comparison. The MS pulp was further processed to produce paper sheets, and the resulting BLs were put through two consecutive stages of precipitation. The raw materials and all liquid and solid samples produced during MSS processing underwent specific chemical characterizations. All experiments were performed in triplicates unless otherwise stated by the mentioned standard methods. The accepted maximum relative standard deviation value was less than 5%.

#### 2.2.1. Pretreatments

Three pretreatments were performed on 300 g o.d. MSS samples: E1 with water, 10:1 liquid-to-solid ratio, 90 min, 75 °C; E2 with pH 12 buffer solution, 10:1 liquid-to-solid ratio, 90 min, 75 °C; and E3 with NaOH solution (8 g/L), 10:1 liquid-to-solid ratio, 90 min, 75 °C. Pretreatments resulted in the formation of three solid phases and three liquid phases.

#### 2.2.2. S_MSS_ Processing via Alkaline Treatment (Soda Pulping)

The S_MSS_ resulting from pretreatments was subjected to soda pulping in order to separate the fibers, along with a fourth sample made up of raw MSSs (untreated). For every solid sample (200 g o.d.), three distinct experimental pulping trials were conducted: PT1 at an alkali charge of 15% NaOH for 30 min at a temperature of 145 °C, PT2 at an alkali charge of 20% NaOH for 30 min at a temperature of 145 °C, and PT3 at an alkali charge of 20% NaOH for 60 min at a temperature of 160 °C. The twelve pulping experiments were denoted as follows: (i) EP1, EP5, and EP9—performed on raw MSSs, following PT1, PT2, and PT3, respectively; (ii) EP2, EP6, and Ep10—performed on MSSs resulting from E1, following PT1, PT2, and PT3, respectively; (iii) EP3, EP7, and EP11—performed on MSSs resulting from E2, following PT1, PT2, and PT3, respectively; and (iv) EP4, EP8, and EP12—performed on MSSs resulting from E3, following PT1, PT2, PT3, respectively. Twelve solid phases (pulp MS) and twelve BLs were produced after the alkaline treatments. Table 1 summarizes the experimental protocol and experiment codes for the MSS biorefinery.

#### 2.2.3. L_MSS_ Processing and Component Separation

The liquid samples from the pretreatment tests were examined for lignin, carbohydrates, and protein using the appropriate techniques (described below). According to the results, the liquid phase produced by E1 was selected for additional processing in order to separate proteins and carbohydrates. In the first processing phase, a rotary evaporator (rotavapor) was used to reduce the liquid extract’s volume to one-third of its initial value. This was followed by a first centrifugation stage that produced a solid fraction (E1C1) and a supernatant (E1C1S1). The E1C1S1 was acidified to pH 4.0 for protein precipitation (the second processing step) and then centrifuged to produce a solid fraction (E1C2) and a supernatant (E1C2S2). In the third processing step, ethanol was added to E1C2S2 (2:1 volume ratio) for hemicellulose precipitation. A solid precipitate, designated E1C3, and a final supernatant, released for ethanol recovery, were the results of the third centrifugation stage.

#### 2.2.4. BL Processing and Component Separation

In order to precipitate lignin, the BLs from pulping trials were first treated with acetic acid until their pH reached 4.5. Centrifugation was used to separate the precipitate, which was subsequently dried and stored for chemical analysis. The resulting supernatants were then utilized to separate hemicelluloses (HCs) using the ethanol precipitation method. Our earlier works [52,53] present more information about the steps involved in the HC separation process. The second centrifugation produced an HC precipitate and a final supernatant, which were released for ethanol recovery.

#### 2.2.5. Solid Sample Processing

The raw MSS and solid samples produced during the L_MSS_ processing were ground and sieved to 0.2–0.5 mm particles before their chemical compositions were determined. After pulping, the solid materials were first cleaned by water washing. Samples weighing 150 g and containing approximately 75% moisture were ground, dried, and analyzed to determine their chemical composition. The pulp samples that showed satisfactory lignin content and sorted yield values (EP9, EP10, EP11, and EP12) were selected and beaten in a Jokro mill (IDM, San Sebastian, Spain) [52]. The beating (2000 revolutions) resulted in pulps of 45–50 °SR drainage resistance. The pulps were further transformed into paper sheets on a Rapid Koethen laboratory sheet former (IDM, San Sebastian, Spain) (ISO 5269/2) [54] in order to observe their potential use as enhancers of the mechanical strength of recycled paper blends including 50% alfalfa stem fiber and 50% fiber recovered from old corrugated cardboard (OCC). The laboratory-produced paper sheets (~70 g/m^2^) were tested to determine their tensile strength (ISO 1924:2008) [55] and burst strength (ISO 2758:2014) [56].

### 2.3. Chemical Characterization: Methods and Assessments

The study took into account the following main components: crude protein, lignin, and structural carbohydrates (polysaccharides). The total structural carbohydrate content or structural polysaccharide (*TSCH*) content was calculated using Equation (1):

(1) $$TSCH(\%)=Glucan\left(\%\right)+Xylan\left(\%\right)+Galactan\left(\%\right)+Arabinan\left(\%\right)+Mannan(\%)$$

The calculations of solid sample carbohydrate contents were based on the concentrations of mono-sugars, sugar recovery standards, and monomer-to-polymer conversion rates.

Cellulose (further referred to as glucan), hemicelluloses, and lignin were determined using the sulfuric acid two-stage hydrolysis method specified by the NREL/TP-510-42618 method [57].

Crude protein was determined by the Kjeldahl method (ISO 5983-2:2009) [58]. A factor of 6.25 was used for conversion from nitrogen content (%) to crude protein content (%).

The following methods were employed to determine the chemical composition of the solid samples in terms of minor components: acetone extractives (AEs)—TAPPI T280 pm-99 standard [59]; hot water extractives—TAPPI T 207 om-88 [60]; and ash—TAPPI T 211 om-02 (2000) [61]. The acid-insoluble lignin (AIL) was determined as a solid remaining on the G3 crucible.

The liquid samples that resulted in both pretreatment and pulping experiments were analyzed for lignin and carbohydrates according to NREL/TP-510-42623 [62]. The total concentration of dissolved solids was determined after water evaporation at 105 °C. This initial processing of the MSS pretreatment liquid samples also resulted in a crude solid extract that was subjected to protein analysis. The ash or inorganic materials were determined by the combustion of samples at 550–600 °C following the specification in NREL/TP-510-42622 [63]. The concentration of organic material was determined by calculating the difference.

The removal rates (*RR*s) of MSS components during various treatments were determined using Equation (2):

(2) $$RR\left(\%\right)=\frac{m_{i}\cdot X_{i}-m_{f}\cdot X_{f}}{m_{i}\cdot X_{i}}\cdot 100$$

where *m_i_* is the initial mass subjected to a specific treatment, *m_f_* is the mass after the treatment, *X_i_* is the initial content, and *X_f_* is the final content of the individual components glucan, xylan, galactan, arabinan, mannan, and lignin (%).

The recovery yields of the HCs and lignin were computed (i) in relation to the amount of the processed MSSs (Equations (3) and (4)) and (ii) in relation to the initial amount of *HC*s or lignin contained in the raw biomass (Equations (5) and (6)).

(3) $${RY}_{L}\left(\%\right)=\frac{m_{L}}{M_{MSS}}\cdot 100$$

(4) $${RY}_{HC}\left(\%\right)=\frac{m_{HC}}{M_{MSS}}\cdot 100$$

(5) $${RY}_{L}^{*}\left(\%\right)=\frac{m_{L}}{M_{L}}\cdot 100$$

(6) $${RY}_{HC}^{*}\left(\%\right)=\frac{m_{L}}{M_{HC}}\cdot 100$$

where *M*_MSS_ is the amount of o.d. MSSs involved in the experiment; *m_L_* is the mass of pure lignin (as AIL); *m_HC_* is the mass of pure *HC*s calculated as the sum of the masses of xylan, galactan, arabinan, and mannan; *M_L_* is the initial amount of lignin in the raw biomass, and *M_HC_* is the initial amount of *HC*s in the raw biomass.

The solid yield (SY, %) was determined by gravimetric means. The solid yield was calculated using Equation (7).

(7) $$SY\left(\%\right)=\frac{m_{0}}{m_{1}}\cdot 100,$$

where m_0_ and m_1_ are the o.d. weight of the solid material before and after the treatment.

### 2.4. Equipment

#### 2.4.1. The Reactor

A 10 L laboratory rotating digester (15 r.p.m.) made of stainless steel and equipped with an electrical heating system and a temperature controller was used for all pretreatments and pulping trials. All experiments were performed at a 10:1 liquid-to-solid ratio. To release the built-up pressure and gather the soda pulp black liquors, the reactor valve was opened at the conclusion of each pulping operation. The resulting BLs were first filtered through a G2 crucible to remove any residual solids. The produced MS pulp was washed and saved for subsequent chemical analysis and processing.

#### 2.4.2. Pulp Processing Gear

The obtained pulps were refined at different revolutions in a Jokro mill. Paper sheets from pure MSS pulp and a blend of 50% pure MSS pulp and 50% OCC were produced using a Rapid Koethen laboratory sheet former. The mechanical strength characteristics (tensile and burst strength) of the obtained paper sheets were measured using a Zwick Roell Z0.5 testing machine (Zwick Roell, Ulm, Germany).

#### 2.4.3. HPLC

A Shimadzu Nexera LC 40D (Shimadzu, Kyoto, Japan) liquid chromatography system equipped with a Shodex SP0810 column (300 × 8 mm, 8 µm particle size, Resonac, Shūnan, Japan) heated at 65 °C was employed to perform the required HPLC analyses. The refractive index detector (Shimadzu RID 20A, Kyoto, Japan) was set at 40 °C. The flow rate of the mobile phase (ultrapure water) was 0.6 mL/minute. The injection volumes were set between 80 and 100 µL, depending on the sample concentrations. Solutions of 99% purity of cellobiose, glucose, xylose, galactose, mannose, and arabinose, provided by Biochem Chemopharma (Cosne-Cours-sur-Loire, France), were used to obtain the HPLC calibration curves in the concentration range of 0.05–2 g/L. Samples, as well as standard solutions, were filtered before injection using 0.2 μm syringe PTFE filters (ISOLAB Laborgeräte GmbH Am Dillhof 2-63863, Eschau, Germany).

#### 2.4.4. FTIR

The FTIR spectra of solids from extracts, hemicelluloses, and purified lignin samples were recorded by using an Agilent Cary 630 spectrometer (Agilent, Santa Clara, CA, USA) in the transmission mode. Sixty-four scans at a resolution of 4 cm^−1^ were performed on KBr pellets with a 1% content of finely grounded samples.

#### 2.4.5. Solid Separation

A Sorvall GLC2 centrifuge (Kroslak Enterprises, Riverview, FL, USA) equipped with an HL-4 rotor, with a CF value of 2012, 3000 r.p.m., was used for solid precipitate separation.

## 3. Results

### 3.1. Chemical Characterization of Raw MS

Three samples, separated from the 2023 harvest, were chosen for chemical characterization: a hay mixture of stems and leaves (MS); a selection of stems (MSSs) and a selection of leaves (MSLs). A fresh harvest (2024) mixture of stems and leaves that had been oven-dried (to prevent any material loss) was also examined for comparison (MS OD). In green harvested alfalfa, the leaves accounted for approximately 60% of the total weight, whereas in field-dried alfalfa hay, they accounted for only approximately about 25%. A number of well-documented factors [34,35,36,37], including mechanical harvesting, mechanical spreading, and gathering during field drying, baling, transport, and storage, have contributed to this sharp decline in leaf content. Given that the majority of the crude protein is concentrated in the leaves, this also significantly reduces the hay’s nutritional value.

The results of the chemical characterization of the alfalfa samples are presented in Table 2. The Supplementary Material’s Table S1 additionally displays the standard deviation for every experiment.

The MSS sample contains the highest amount of cellulose of all samples (expressed as glucan in Table 2). Cellulose represents the major part of the polymeric carbohydrates (61.9%), while HCs accounted for about 20% of the MSS sample weight, the equivalent of 38% of polymeric carbohydrates. Xylan is the most important representative of the HCs in MSSs, accounting for about 61% of their total mass. About a fifth of the MSS sample’s oven-dried weight was made up of lignin, which was relatively higher (about 50%) than that of other biomass resources like corn stalks and wheat straws [52,64]. The MSS protein content was only 8.54%, while in the MSLs, the protein content was close to a quarter of the sample weight (24.11%). This demonstrates the importance of the stem-to-leaf ratio in protein extraction from MS and, more broadly, in MS bio-refineries. As with MSSs, approximately one-fifth of the oven-dried weight of the MSL sample was composed of lignin. About 20% of the MSL sample is made up of TSCH, of which slightly more than half is made up of cellulose. Arabinan was found to be the primary non-cellulosic polysaccharide in MSLs, making up 38% of all HCs.

Generally, the reported experimental results correlate well with literature data [23]. Nonetheless, some distinctions can be observed with regard to alfalfa’s chemical constituents. In this regard, Zhou et al. recently reported a slightly different cellulose and xylan content (glucan 20.6%, xylan 9.9%) for the green harvested hay samples, despite similar protein content [65]. Dien et al. [66] reported a 48.9–51.3% polysaccharide content for o.d. MSSs harvested in the green state. The AIL determined by the Klason method ranged from 11.9 to 14.6%, while the protein content ranged from 12.2% to 13.2%. Among the structural carbohydrates, cellulose accounted for 31.1%, whereas in certain samples, xylan was at 15.5%. The content of Arabinan was found to be a mere 0.3%. The chemical composition of MSSs is greatly influenced by cultivar, harvesting time and schedule, irrigation, and water salinity, as demonstrated by Warnke and Ruhland [67]. According to their analysis, the cellulose content ranged from 35% to 37%, while the HC content varied from 8% to 10%. The lignin content was estimated to range between 18% and 19%. The chemical composition of MSSs was reported by Lamb et al. [68] to be 29.2% cellulose, 8.6% xylan, and up to 2% galactan, mannan, and arabinan.

Our findings and data from the literature indicate that the leaves concentrate the crude protein. The typical range of leaf protein is from 18% to 26%, and it depends on the cultivar, harvesting time, irrigation, and other field conditions [69,70].

### 3.2. Chemical Characterization of the Solid Phase Resulting After Pretreatments (S_MSS_)

The pretreatment effect on the chemical composition of S_MSS_ is presented in Table 3. The standard deviation values are shown in Table S2. An immediate observation is that glucan content increases with the pretreatment harshness. The HC content suffers a considerable drop from about 20.3% in raw MSSs to 13.4% after E1, 15.04% after E2, and 16.4% after E3.

The protein content of the pretreated samples varies within a narrow range from 5.67% to 6.67%. The loss of the other components during the pretreatments caused the increase in lignin content in the solid phase.

**Figure 3:**
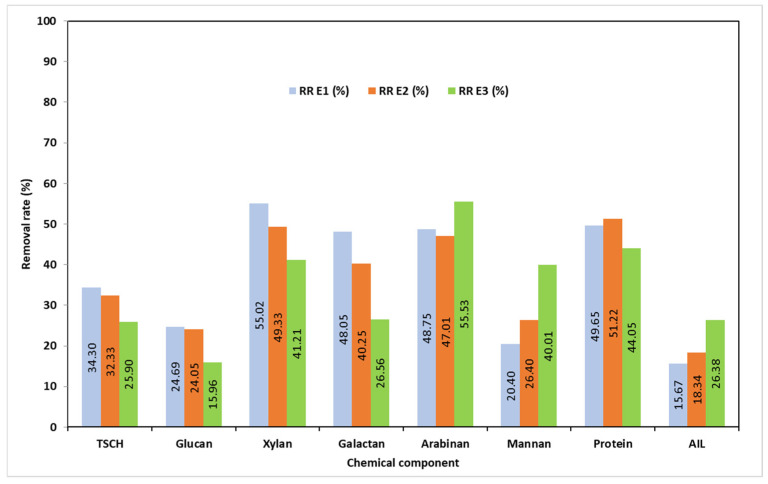
The removal rates (RRs) of chemical components during MSS pretreatments.

As for the pretreatments’ efficiency in terms of removal rates, the results are presented in Figure 3.

Water pretreatment (E1) had a significant effect on the majority of the chemical constituents of MSSs, resulting in the removal of 34.3% of the structural carbohydrates (24.7% of glucan, 55% of xylan, and consistent amounts of arabinan, galactan, and mannan). The protein content was reduced by 49.6% after E1. With a removal rate of only 15.67%, E1 had less of an impact on acid-insoluble lignin than either E2 (18.34%) or E3 (26.38%). A significant decrease in mineral content—82% ash—may also be observed with this kind of treatment. The acid-soluble lignin content varied by 45.3%.

Compared to water pretreatment (E1), the pH12 buffer solution pretreatment (E2) eliminated fewer carbohydrates. Glucan removal rates for E1 and E2 are fairly comparable. Compared to E1, the removal rate of xylan was found to be lower, and the same findings apply to arabinan. For E2, the rate of mannan removal was higher. For the crude protein removal rate, there is a slight difference in favor of E2. The AIL removal rate rose when moving from E1 to E2, but the AIL RRs remained relatively constant. A 60% reduction in ash content was attained using E2.

At last, the E3 pretreatment shows the least amount of influence on structural carbohydrates like galactan (~16%), xylan (41.2%), and glucan (25.56%). E3 was the least successful in removing crude protein out of all the treatments, but it had the highest rates of mannan and arabinan removal (removal rate of 44%). Regarding the ash content, a removal rate of roughly 80% was noted.

### 3.3. Chemical Characterization of the Liquid Phase Resulting After Pretreatments (L_MSS_)

The effects of each type of pretreatment on the chemical composition of L_MSS_ can be appraised from the data in Table 4 (standard deviation presented in Table S3).

The chemical composition of the L_MSS_ liquors included carbohydrates, lignin, and protein. Carbohydrates made up roughly 21% of the dissolved organic matter (OM) in the E1 liquid phase, 17% of the OM dissolved in the liquid resulting from E2, and 22% of the OM in the E3 liquor. The concentration of xylan residues, which are the primary representative of the carbohydrates in all liquors, rose from E1 to E3, from roughly 36% in E1 to 49% in E3. The concentration of lignin increased when the extraction’s pH was changed from E1 to E3. In organic matter, the lignin content rose from 7.85% in E1 to 13% in E3. These changes are a direct result of the pH value rising from E1 to E3. The protein content of the solid residue that was recovered following water evaporation showed a strong correlation with the protein removal rates calculated from the SMSS protein content. Because the water extraction (E1) produced the highest protein content in both dissolved solid material and OM, the corresponding liquor was selected for further processing through centrifugation, pH reduction, and ethanol treatment (Figure 2).

Data from the literature on lignocellulosic biomass pretreatments indicates that LMS stems are more susceptible to the applied treatments than corn stalks or wheat straws. Therefore, compared to the materials mentioned above, LMS stems have a tendency to release their chemical components under far milder conditions. According to Serna-Loaiza, et al., hot water treatment of wheat straw at 160 °C for 90 min resulted in a mass loss of roughly 20%, while the resulting liquor contained 2.15 g/L of oligomers resulting from xylose and xylan [71]. The sugar concentrations for the wheat straw hydrothermal treatment liquid reported by the same authors also fall within similar ranges, considering that the authors used similar liquid-to-solid ratios [72]. Similar findings hold true when comparing MSSs to corn stalks, which require far harsher conditions in order to release comparable amounts of carbohydrates [73,74].

### 3.4. Chemical Characterizations of the Solids Obtained by Sequential Fractionation of E1 L_MSS_

**Figure 4:**
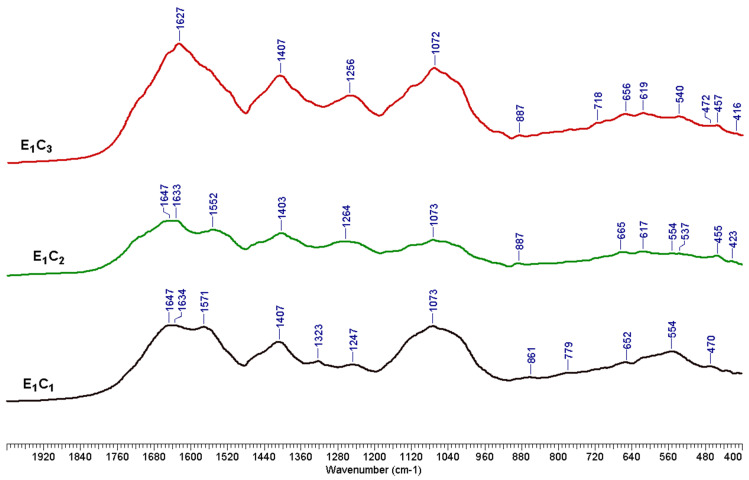
The FTIR spectra of solids isolated from E1 L_MSS_.

As shown in Figure 2, the LMSS produced following the water pretreatment (E1) was successively fractionated to yield three different types of solid materials (precipitates), designated E1C1, E1C2, and E1C3 (see Section 2.2.3). Table 5 and Table S4 present the chemical composition of these solid fractions, while Figure 4 displays the corresponding FTIR spectra.

The E1 LMSS was fractionated using ethanol treatment, pH reduction, and liquor evaporation. Three protein fractions with comparatively high protein content were obtained. With xylan making up around 4% of each product, the carbohydrate content was comparatively low. The highest amount of carbohydrates was found in E1C3 (16.55%). While the lignin content decreased from nearly 18% to roughly 15%, this precipitate displayed a higher amount of arabinan. For E1C2, the protein content reached approximately 44%, whereas for E1C1 and E1C3, the protein content accounted for 48% and 51% of the total components identified. According to the results thus far, water treatment might be a viable strategy for protein extraction from MSSs.

The stretching vibrations of the O-H bond in phenolic and alcoholic hydroxyls produced a wide absorption band at about 3400 cm^−1^. Peaks at about 2920 and 2840 cm^−1^ are the results of the C-H stretching vibrations of methyl and methylene [75]. The peaks occurring at 1647 and 1550 cm^−1^ are specific protein bands. The E1C1 and E1C2 samples clearly show the 1647 cm^−1^ peak, but the E1C3 sample spectra only show a shoulder because of the overlap caused by the presence of other components like lignin and carbohydrates. The amide I band is usually identified by this peak, which is created by the peptide bond’s C=O stretching vibrations [76]. The N-H bending vibration and the C-N stretching vibration are the causes of the peak at 1550 cm^−1^, which is identified as the amide II band. The amide III band, which is the result of C-N stretching vibration and N-H bending vibration, was also visible in all spectra at absorbance maxima of about 1240 cm^−1^ [77,78]. The peak around 1400 cm^−1^ indicates the presence of protein side-chain carboxylate COO-. Every spectrum showed peaks at about 3300 cm^−1^, which were ascribed to N-H bending vibration [79,80].

### 3.5. Chemical Characterizations of the MS Pulp Obtained After Pulping Trials

The solids resulting from pretreatments, along with a fourth raw MSS sample, were subjected to a series of three alkaline treatment experiments (see Section 2.2.2).

#### 3.5.1. Chemical Characterizations of the Solids Obtained After PT1

The effects of PT1 on the chemical composition of the resulting pulp can be assessed from the data in Table 6 and Table S5.

Glucan and xylan are the primary structural carbohydrate constituents of the solid materials that emerged following the PT1. The lowest solid yield is achieved for the raw MSSs. The E1–PT1 and E2–PT1 combinations yielded the highest SYs with comparable values, nearly 60%, whereas the E3–PT1 combination yielded a slightly lower SY (55.47%). According to the AIL (%) values, the lignin content appears to be unaffected by the pretreatment conditions. A similar resistance to alkali treatment was documented by Chen et al. in relation to an alkali treatment (5% NaOH) that caused only minor decreases in the amount of lignin in treated alfalfa hay [81].

**Figure 5:**
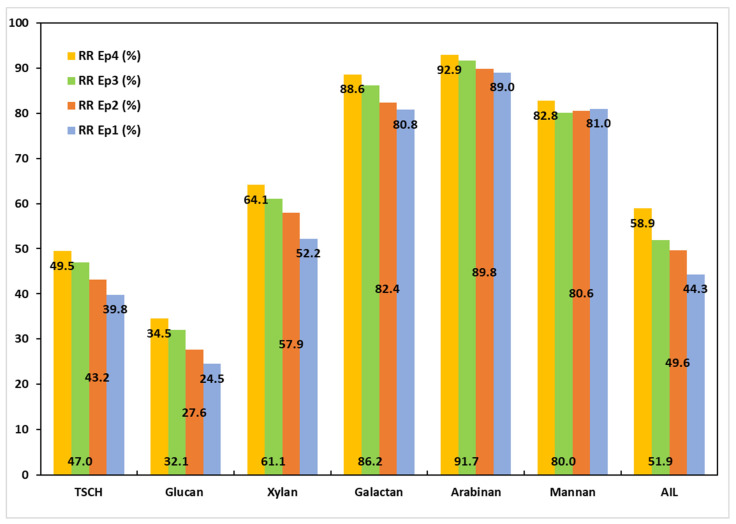
Removal rates of MSS components following the PT1 trials.

Figure 5 shows the removal rates for each component, providing a clearer picture of the impact of the combinations of pretreatment and the PT1 process. The data presented in Figure 5 show that the raw material MSSs have the lowest loss of carbohydrates at 39.8%. The carbohydrate loss in the pretreated samples increases as the severity of the pretreatment increases in the following order: E1 < E2 < E3 (43.2%, 47%, and 49.5% respectively). The individual components also show a similar trend, with a higher impact for removal rates of glucan, xylan, and galactan. The arabinan and mannan RRs appear to be less affected by the pretreatment type. In the case of lignin removal, the impact of pretreatment is more evident. There is a notable difference between the lignin RRs of the MSSs that received alkaline pretreatment (E3—58.9%) and the raw material (without pretreatment—44.3%). The process selectivity was generally low, regardless of whether MSS was untreated (raw) or pretreated; the ratios of the lignin removal rates to the total carbohydrate removal rates ranged from 0.84 to 0.91, and these values are fairly comparable.

#### 3.5.2. Chemical Characterizations of the Solids Obtained After PT2

**Figure 6:**
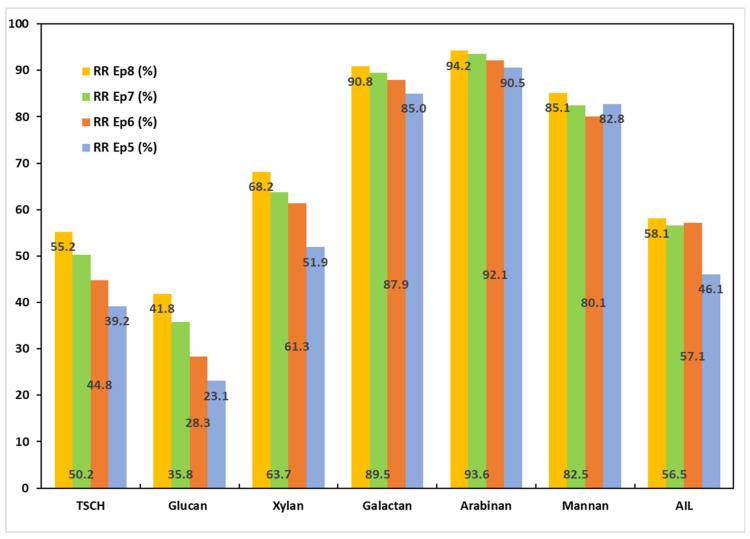
Removal rates of MSS components following the PT2 trials.

The data in Table 7 and Table S6 presents how PT2 affected the final pulp’s chemical composition. A better understanding of the effects of the combinations of the pretreatment procedures and the PT2 process can be found in Figure 6, which displays the removal rates for each component. The solid yield for the raw MSSs is slightly lower after PT2 than after PT1. The total carbohydrate content of the solid samples obtained after PT2 was generally higher, but the acid-insoluble lignin content stayed between 20% and 22%. The lignin recalcitrance was not broken by the alkali charge increase alone.

The alkali treatment at 145 °C and a 20% NaOH charge (PT2) resulted in an increase in all removal rates for every MSS component. The increase was between 4% and 10% in magnitude. While the losses for the water-treated MSSs (Ep6) varied by only 1.6%, the total structural carbohydrate losses for the control trial (EP5) did not vary at all. Further increases of 3.2% for EP7 and 5.7% for EP8 could be observed for the remaining samples. The components that were removed the most were mannan, arabinan, and galactan, just like for PT1 trials. The rates of xylan and glucan removal increased by 2–20%. Compared to the PT1 results, the lignin removal rates increased by 4% in EP5, 15% in EP6, and 8.9% in EP7. The ratio between the removal of lignin and carbohydrates did not rise much; it was between 0.78 and 0.95, with EP6 having the highest value.

#### 3.5.3. Chemical Characterizations of the Solids Obtained After PT3

**Figure 7:**
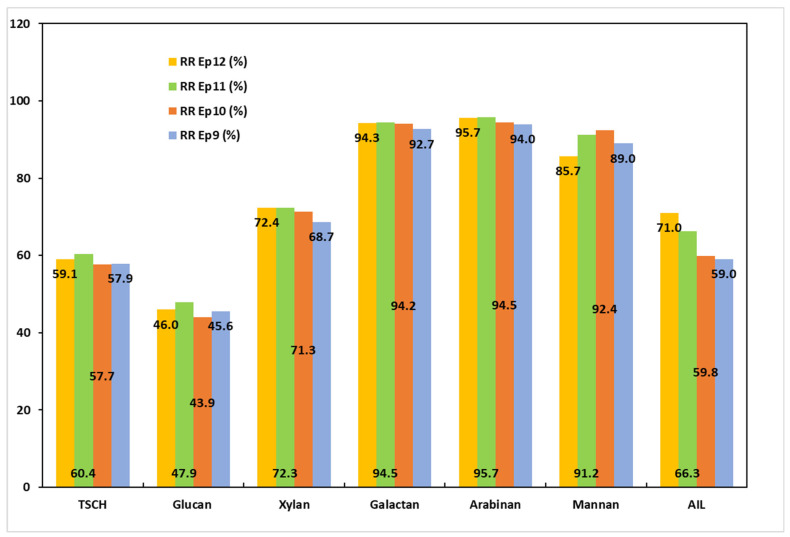
Removal rates of MSS components following the PT3 trials.

The information required to evaluate the effects of the combinations between the pretreatment procedures and PT3 is provided in Table 8 and Table S7 and Figure 7.

The third set of tests evaluated the reaction of MSSs, both raw and pretreated, to pulping with a 20% NaOH alkali charge for 60 min at 160 °C. The pulping conditions’ significant escalation in severity led to the lowest overall yield values. In all of the samples, the amount of total structural carbohydrates was decreased to less than 65%. In every experiment, their removal rates exceeded 55%. When compared to the earlier experimental series (PT1 and PT2), EP9 (raw MSSs) showed the most significant increase, with a variation of more than 40%. When the temperature and process time are increased by a factor of 1.1 to 1.8, the removal rate also increases in tandem with the increase in the glucan content. Compared to PT2, the xylan removal rate rose from 6% to 32%. AIL removal rates were also higher than for PT1 and PT2, despite the apparent lack of the delignification effect of increasing temperature and pulping time. The ratio of the AIL removal rate to the carbohydrate removal rate was determined to be between 0.83 and 0.98. As the treatment becomes more severe, the solid yield values obtained in the pulping trials continue to decline. These findings are similar to those of Ai and Tschirner [48]. The lower sorted yield values are a significant disadvantage that the earlier authors also noted and that might impede the potential use of alfalfa stems as a virgin fiber source. Nevertheless, only the pulps from PT3 were selected for papermaking, based on the chemical composition results.

### 3.6. Chemical Characterizations of the BLs Obtained After Pulping Trials

Pulping is an alkaline biomass treatment technique that primarily aims to liberate cellulose fibers from their hemicellulosic and lignin matrix for papermaking. It produces a significant amount of residual liquid known as black liquor (BL). The relationship between pulping process parameters and their influence may become clearer after an analysis of the BL chemical composition. Additionally, this analysis might offer vital details regarding possible pathways for the extraction of valuable substances like lignin and dissolved hemicelluloses. In the Supplementary Materials, in Table S8, the main findings regarding the chemical composition of the black liquor samples produced following both raw MSS and SMSS pulping are presented. Several remarks can be made in light of the raw data shown in Table S8. Firstly, there is less organic matter in black liquor produced from pretreated LMS stems. Higher amounts of organic material are released when the alkaline treatment’s severity (temperature, alkali charge, and time) increases. While some samples did contain mannan, the amount in black liquor was minimal and challenging to measure. Nevertheless, the recovered HC samples had a mannan content of 1–2%.

**Figure 8:**
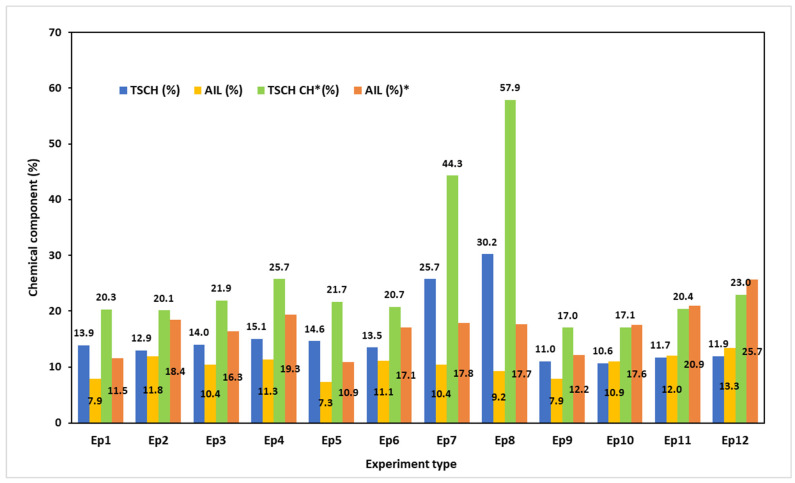
Distribution of polymeric carbohydrates and lignin in the chemical composition of the BLs (reported for SM, * reported for OM).

The distribution of the polymeric components, including lignin and total structural carbohydrates, in relation to both the solid matter (SM) and the organic matter (OM) of the black liquor is shown in Figure 8.

According to Figure 8, lignin and carbohydrates comprise up to 41% of the dissolved components in the black liquor, while they account for up to 60% of the dissolved organic matter. Both process conditions and sample types affect the ratio of TSCH (%) to AIL (%). The raw MSS pulping at 145 °C (PT1 and PT2) resulted in a BL with dissolved organic material contents of 20.3% TSCH and 11.5% AIL (EP1) and 21.7% TSCH and 10.9% AIL (EP5). Increases in alkali charge (PT3) facilitate the dissolution of carbohydrates. The TSCH percentage decreases from 21.7% (EP5) to 17% (EP9) as pulping conditions become more severe (160 °C and 60 min pulping time). This causes the TSCH (%)-to-AIL (%) ratio to fluctuate, with calculated values ranging from 2.0 to 1.4. In the case of solids resulting from pretreatments (SMSS), the situation is different. The water pretreatment (E1) appears to cause a slight alteration in the TSCH (%) while promoting a rise in the AIL (%), and therefore the ratio of TSCH (%) to AIL (%) experiences minor alterations. The BLs produced by S_MSS_ pulping were found to have comparable chemical composition to other pulping processes documented in the literature. In the case of wood mixtures (33% softwood and 67% hardwood), Zhu et al. found that BL had a total dissolved solid content of 33.2%, with AIL being the predominant fraction (80%). Xylan residues made up approximately 50% of total structural carbohydrates (8%) [82]. It should be noted that the pulping solid-to-liquid ratio, as well as the process parameters and the nature of the raw biomass, have a significant impact on the chemical makeup of the solid fraction of BL [83]. Using eucalyptus wood as a raw material, Sharma et al. found that Kraft pulping black liquor contained approximately 14 g/L of xylan [84].

The chemical composition and distribution of the organic components of the BL produced by S_MSS_ soda pulping were affected by the pH variations in the pretreatments, determining the increase in both TSCH (%) and AIL (%). The increase in alkali content from PT1 (15% NaOH charge) to PT2 (20% NaOH charge) along with pulping time and temperature caused the doubling of the TSCH (%) found in the organic material but had no noticeable effect on the AIL (%). Furthermore, the ratio of TSCH (%) to AIL (%) varies significantly, with calculated values ranging from 2.5 to 3.3. In these circumstances, between 62% and 75% of the dissolved OM in black liquor is made up of lignin and carbohydrates. Conversely, if the alkali charge is kept at 20% and the pulping intensity is raised, the amount of sugars and lignin in the OM decreases. The observed effects can be explained by the overlap between the processes that remove lignin and dissolve and degrade polymeric carbohydrates [85,86]. The dissolution of the carbohydrates is accelerated by the increase in NaOH concentration (and consequent increase in alkali charge at a constant solid-to-liquid ratio) in pulping liquor. Both the solubility of HCs and the cleavage of the lignin carbohydrate linkages are impacted by the concentration of NaOH [87,88].

The comparison attempt encounters some obstacles, which can be attributed to variations in raw materials, pulping conditions, or other elements like the analysis methods. According to Morya et al., the total dissolved organic matter in agricultural residues that result in BLs is approximately 8–18% TSCH and 28–32% AIL [89]. The results of the current study fall within the aforementioned ranges.

### 3.7. Characterization of the Recovered Hemicelluloses

**Figure 9:**
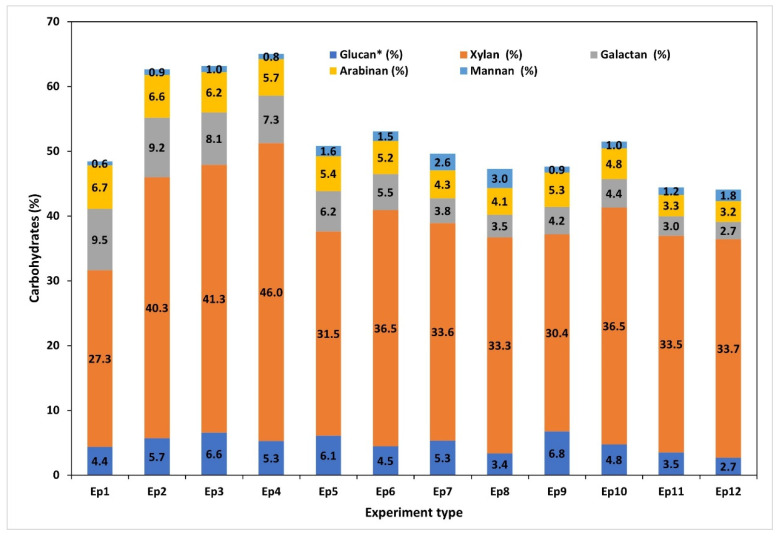
Chemical composition of separated hemicelluloses samples (HC’s) showing variable contents of xylan and other polysaccharides as a function of experiment type.

The characterization of hemicelluloses extracted from the black liquor of MSS pulping was performed in order to ascertain their chemical composition, which is displayed in Figure 9, as well as how the MSS pretreatment conditions affect the chemical composition. The chemical composition of separated hemicellulose samples (raw HCs) is presented in Table S9, in the Supplementary Materials. Xylan was the primary polymeric carbohydrate in all of the samples, with contents ranging from 27% to 46%. The liquor produced by soda pulping at a low alkali concentration (15%) allowed for the recovery of HCs with the highest xylan content and highest purity (62–65%), expressed as the sum of the total structural carbohydrate content. The data for the raw MSSs and pretreated MSSs show that the preliminary extraction has a positive effect on the HC composition. The ratio of xylan to arabinan in the HC extracted from black liquor varied from 4 to 10, depending on the pretreatment employed and the pulping parameters. In comparison to the HCs recovered from samples that underwent pretreatments, the BL recovered from raw (untreated) MSS exhibits lower xylose–arabinose ratios. The ratio of xylan to arabinan also increased when the temperature or alkali charge increased.

The chemical composition of isolated HC samples can be compared with literature data. The amounts of xylan in the MSS HC samples are comparable to those of HCs recovered from wood and non-wood black liquor. The xylan amount of an alkali-extracted HC sample ranges from 14% to 85%, according to Zhang et al., who also noted how the process variables affect the xylan content [90]. Using a more complicated separation process, Sharma et al. achieved higher values (60–70%) for the xylan content of HC samples recovered from Kraft BL of eucalypt wood [84].

**Figure 10:**
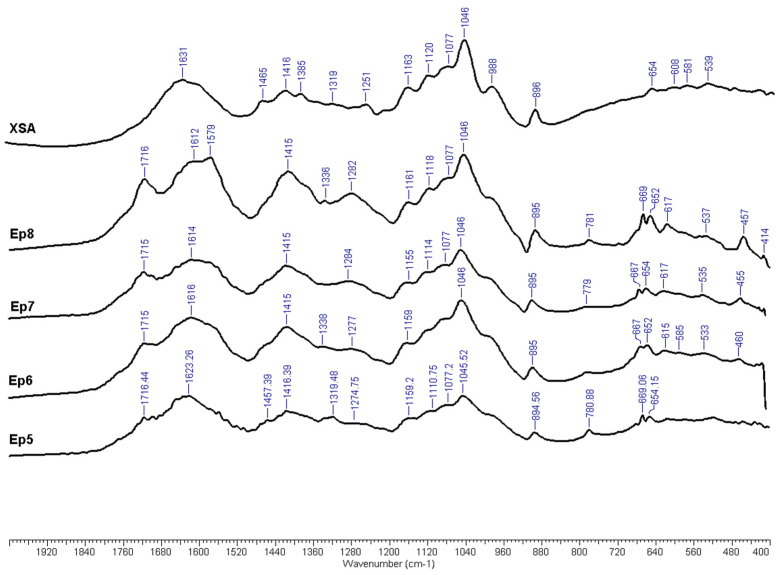
FTIR spectra of isolated HC samples (selection).

Figure 10 displays a number of representative spectra of the acquired HC samples, while Figure S1 presents the FTIR spectra of isolated HC samples for all experiments. The stretching vibrations of the O-H bond in alcoholic hydroxyl caused a broad absorption band at about 3400 cm^−1^ in all of the HC samples that were examined. The methyl and methylene C-H stretching vibration resulted in peaks occurring at ~2930 and ~2850 cm^−1^ [91]. There are several bands in the 2000–800 cm^−1^ range. Aldehyde carbonyl group stretching causes bands that appear at about 1715 cm^−1^. These bands are primarily found in Ep5–EP12 samples, and their intensities typically increase as the severity of alkaline treatments increases. The band at 1610 cm^−1^ that corresponds to the presence of lignin as an impurity is overlapped by the broad band at 1650 cm^−1^ that is linked to the presence of water as moisture [92]. Additionally, the presence of lignin is demonstrated by shoulder or low-intensity sharp bands at about 1505 cm^−1^. The 1278 cm^−1^ band can also be attributed to the lignin impurity [93]. The presence of the amide II deformation low-intensity bands at 1550 cm^−1^ in some of the samples may also indicate potential protein contamination. The moderately intense band occurring at ~1415 cm^−1^ was attributed to COO- groups [94]. The bands occurring in the range 1250–1140 were assigned to the stretching vibration of the C-H in carbohydrates [95], while the bands occurring at ~1110 cm^−1^ were assigned to C-O stretching [94]. Ottah et al. suggested that bands occurring at ~1160 cm^−1^ are a result of the asymmetric stretch of asymmetric β-1, 3-glycosidic bonds in gluco-mannan chains [96]. Furthermore, the ~1075 cm^−1^ bands may be attributed to secondary OH stretching in carbohydrates [97]. The band occurring at ~1046 cm^−1^ was assigned to C–O stretching vibration in polymeric carbohydrates [98]. Although the ~890 cm^−1^ band is associated with C1–H bending in xylose residues in xylan by some authors [99], this band is more often associated with interunit C-O-C glycosidic linkage [100].

### 3.8. Characterization of the Recovered Lignin

**Figure 11:**
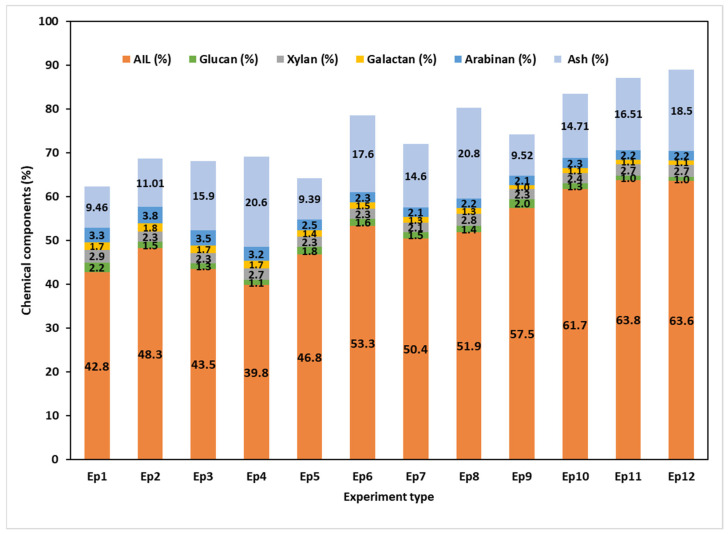
Chemical composition of separated lignin samples showing variability of purity as AIL as function of experiment type. The different content of carbohydrates is also visible.

The chemical composition of the separated soda pulping lignin samples is displayed in Figure 11 and presented in Table S10, in the Supplementary Materials (standard deviation included). All samples that were isolated by acid precipitation contained both lignin and carbohydrates that co-precipitated during the separation, either as hemicelluloses or, more likely, as lignin carbohydrate complexes (LCCs), as some authors have described [82,101,102,103,104]. Typically, the separated lignin samples had a TSCH content between 7% and 10%. The amount of carbohydrates in the isolated lignin samples appears to be slightly influenced by the severity of the pulping process and less affected by the pretreatment conditions. The AIL contents of the separated lignin samples ranged from 42.8% to 63.8%. The AIL value reflects the impact of the pretreatments and the soda pulping experimental parameters on the samples’ purity. Regarding the raw MSSs, the lignin purity increased by just 9.3% as a result of changing the alkali charge from 15% (PT1) to 20% (PT2). Additional temperature and alkali charge increases (PT3) resulted in a 34% increase in lignin purity. A further analysis of the effects of pre-extraction stages at a pulping temperature of 145 °C and an alkali charge of 15% revealed that the water extraction (E1) had the biggest effect on lignin purity.

Another indication that water extraction has the biggest effect on lignin purity is the rise in alkali charge at the same temperature (PT2). However, the temperature increase at a 20% alkali charge (PT3) causes a shift in the hierarchy of impacts; the extraction with alkali (E3) and pH12 buffer solution (E2) produced the highest-purity lignin. Although raw material species and pulping conditions may vary, the reported values for AIL (%) are comparable to those in the literature. In this regard, Zhu et al. [82] found that lignin recovered from BL from wood (a blend of softwood and birch) had an AIL content of 32–62%, while Tiller et al. showed that raw lignin recovered from Kraft pulping BLs of Korean oak, acacia, and softwood [105] contained 60–70% AIL. In the case of the non-wood pulping, raw lignin recovered from black liquors may contain 44–60% AIL for Miscanthus soda pulping BL [106], 50 to 65% for rice and wheat straw soda pulping [107,108,109], and 20–50% for lignin isolated from the BL of soda pulping of bamboo chips [110]. The purity of commercially available lignin products may vary between 75 and 98% [111].

**Figure 12:**
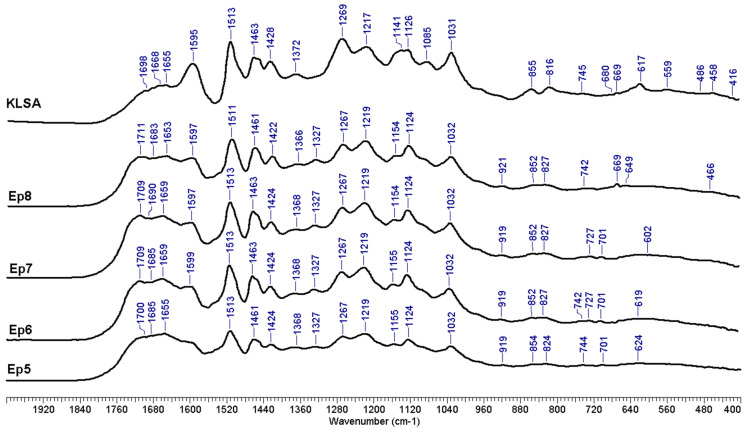
FTIR spectra of isolated lignin samples (selection).

A selection of FTIR spectra for isolated lignin samples is displayed in Figure 12, while Figure S2 presents the FTIR spectra for all purified lignin samples. The chromatogram of the standard mixture of calibration sugars is shown in Figure S3, and the chromatogram of the sugars detected in MSS hydrolysate is shown in Figure S4.

A broad absorption band at about 3400 cm^−1^ was seen in all of the recorded lignin spectra, which was caused by the stretching vibrations of the O-H bond in phenolic and alcoholic hydroxyl. Peaks at approximately 2930 and 2850 cm^−1^ were produced by the C-H stretching vibration of methyl and methylene [112]. The existence of C=O in aldehydes that may be present in saturated side chains of lignin is what causes the ~1740–1710 cm^−1^ band [113]. In some samples, peaks at ~1680 cm^−1^ are clearly observable and may be attributed to conjugated carbonyl group stretching. The ~1460 cm^−1^ peak presence may be associated with the asymmetric bending of the methyl and methylene [114]. Furthermore, the ~1420 cm^−1^ peak occurs as a result of the C-H in-plane deformation vibration associated with aromatic ring stretching. The aromatic skeletal vibrations bands are observable at ~1603 and 1513 cm^−1^ [115].

The peak present at ~1370 cm^−1^ may be assigned to the symmetric C-H bending of the methyl groups in methoxy groups [116]. According to Sammons et al. [117], the peaks of the S-ring are revealed by the presence of the ~1330 cm^−1^ peak (C-O stretching), while the presence of the G-type ring is evidenced by ~1265 cm^−1^ absorption maxima. The 1160 cm^−1^ peak indicates that the studied lignin type is a HGS lignin [118]. The peak at ~920 cm^−1^ may be assigned to C-H vibrations [119]. According to Shi et al. [120], the presence of the absorption at ~829 cm^−1^ should be assigned to C-H out-of-plane vibrations in H-type lignin units. The bands occurring in the range 1210–1230 cm^−1^ may also be assigned to various stretching vibrations of either C-C, C-O, and C=O, as indicated by Negrao et al. [121]. Finally, the peaks occurring in the range 1080–1030 cm^−1^ may be associated with the various vibration modes of the C-O in alcohols and phenols [122,123].

### 3.9. Hemicellulose and Lignin Recovery Yields

**Figure 13:**
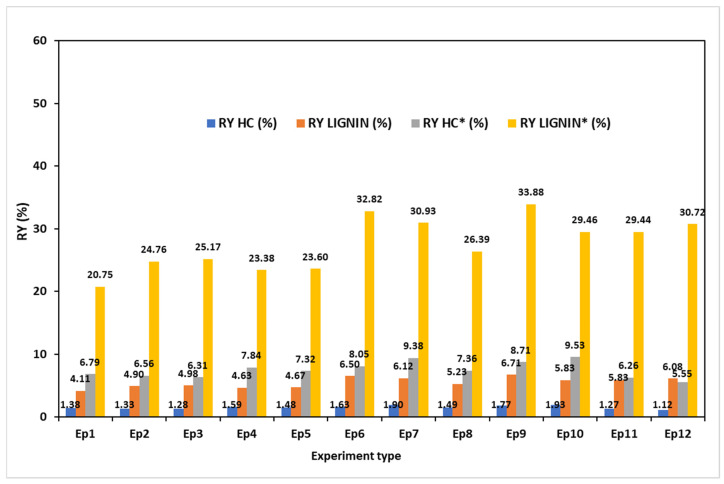
Hemicelluloses and lignin recovery yields values in dependence on experiment type and in computed relation with the total amount of biomass and, with the amount of the HCs and lignin (as AIL) contained by the MSS (* marked).

Figure 13 displays the values of the HCs and calculated recovery yields (RYs) (i) in relation to the initial amount of MSSs and (ii) in relation to the initial amount of HCs in the raw MSSs. With the exception of tests EP6 and EP7, where the E1 and E2 pretreatments had a beneficial effect, it is evident that extractive pretreatments have a negative impact on the HC recovery yields. The increasing severity of soda pulping in terms of both alkali and temperature stimulates the dissolution of carbohydrates, resulting in a rise in the HC recovery yield. However, the most severe pulping conditions produced the lowest values of the HC recovery yields, which can be explained by HC degradation in the pulping reaction environment. The current results are generally lower when compared to data from the literature. For instance, Teramura et al. found that the HC recovery after treating sorghum bagasse was roughly 13% [124]. Similar values were reported by Johakimu et al. for Eucalyptus grandis wood biomass [125]. Higher values of roughly 20% were recorded by Ottah et al. for HC extraction from wood [96] and by Dafchahi and Acharya for HC extraction from wheat straw [126].

The lignin recovery yield values appear to be positively influenced by water pretreatment (E1) and lower-temperature soda pulping. The EP2 and EP6 treatments increased the RY by 19% and 39%, respectively, when compared to untreated MSS pulping. Higher temperatures and pulping time reduce the lignin RY by approximately 13%. This decline in lignin RY was linked to the dissolved lignin’s deterioration as pulping time and temperature increased.

The data presented in the literature exhibits significant variation with respect to process conditions, biomass, and reporting format. Nevertheless, the lignin values revealed in this study are similar to those for lignin recovery from corn biomass reported by Zhang et al. [127], which ranged from 21.2 to 57%. Lu et al. reported values of lignin recovery yields in the range 41–76% [128]. Similar ranges of values (28–40%) were noted for the alkali lignin found in sugarcane waste [129]. Zheng et al. obtained values for the recovery of lignin from wheat straw organosolv residual liquors that ranged from 30% to 55% [130].

### 3.10. The Papermaking Potential of the MSS Isolated Fibers

To explore the possibilities of MSS fibers in papermaking or as blends for recovering the strength of lost recycled fiber, paper sheets were made using both pure MSS fibers and a blend made of 50% MSS fibers and 50% fiber recovered from OCC. In terms of tensile and burst strength, MSS fiber showed promising results, improving values by up to 35–36% for tensile strength and 70% for burst strength. The findings of the studies on the potential for papermaking of both single MSS fibers and blends of MSS and OCC recycled fibers are shown in Table 9 and Table S11.

#### 3.10.1. Pure MSS Fibers

The tensile index values of the majority of the samples are around 20% lower than those reported by Ai and Tschirner [48]. However, as the same authors observed, the MSSs responded well to bleaching, and the tensile strength nearly doubled following bleaching, supporting the idea that the lignin content and chemical composition are related to paper strength. The MSS paper sheets behaved similarly in our experimental setup. Both tensile and burst strength were generally improved by the pre-extraction of proteins. The water pretreatment (E1) was the most advantageous from the perspective of the tensile index, as evidenced by a 23% increase in tensile strength compared to control sample Ep9 (raw MSSs). The pretreatment with 8 g/L NaOH (E3) resulted in the greatest improvement in burst strength.

Because the authors of [48] do not specify whether or not the pulps were beaten in a laboratory, it is challenging to make further comparisons. Nevertheless, in terms of burst strength, the paper composed of individual MSS fibers has a relatively low value. Ai and Tschirner [48] reported a burst strength of about 2.85 kPam^2^/g. When compared to other fiber sources discussed in the literature [131], fibers from MSSs are at least equivalent to those from wheat straw (although the stated values for the tensile index are slightly wide) For instance, Urdanetta et al. reported values of around 27 N·m/g [132], while Liu et al. reported values of approximately 80 N·m/g [133]. Furthermore, the MSS results are similar to those of cellulosic fibers isolated from corn stalks. Jahan and Rahman reported a drainage resistance of about 30°SR and a tensile index values value of 60 N·m/g at 55°SR for corn stalk pulp [134].

Although various potential uses of non-wood fibers in paper formulations, including tissue paper, were reported [135], the main application of non-wood fibers remains the production of packaging paper [136,137,138], and the paper samples reported in our work present the necessary characteristics for this use.

#### 3.10.2. Blend of 50% MSS–50% OCC

Non-wood fibers, especially agri-waste-derived pulp, are often mentioned as a potential reinforcement for recycled paper. Additionally, a lot of authors acknowledge that paper packaging offers a flexible, environmentally friendly, and frequently economical way to display and preserve goods. Paper recycling is known to naturally shorten and weaken cellulose fibers, which lowers the strength of the final product. A number of studies tackle this performance, although their primary focus is on straw pulps and secondary fiber blends. Mayank et al. [139] recovered the strength loss of recycled softwood pulp by using different ratios of straw pulp. They demonstrated that adding more wheat straw pulp to the papermaking blend greatly increased the tensile index. Other studies highlight the possible application of non-wood fibers in the synthesis of cellulose nanomaterials. Such materials proved their value as strength boosters for packaging paper [140].

Hagel and Schütt explored the potential usage of fibers separated from wheat straw as a method to increase the mechanical strength of recovered paper [141]. They concluded that the presence of 30% wheat straw fibers in recycled paper formulations may not necessarily improve the strength. However, recent research suggests that wood and non-wood fiber mixtures could be utilized in papermaking formulations with positive outcomes. In this regard, the research by Serna-Loaiza et al. revealed a number of suitable possible formulations that contained up to 30% non-wood pulps [64].

According to the current investigation, whether raw (EP9) or pretreated (EP10, EP11, and EP12) MSS fibers were combined with OCC, all monitored parameters (tensile index, burst strength, and drainage resistance) increased in comparison to CCR values (Table 8).

## 4. Conclusions

In this study, a possible route for the biorefinery of *Medicago sativa* biomass was proposed in order to produce multiple valuable outputs: cellulose (glucan), lignin, hemicelluloses (xylan, galactan, arabinan, and mannan), and a protein extract. The starting raw material was constituted by one-year-old, field-dried, balled alfalfa hay, inappropriate for feed/food use and thus considered as agricultural waste. During experiments, several key aspects were investigated: the impact of the pretreatments, the impact of the pulping parameters (pH, temperature, alkalinity, and process duration), and the raw material composition. All liquid and solid outputs were thoroughly investigated for their chemical composition in terms of polysaccharides and protein content. Furthermore, the cellulose fibers generated from MS stem pulping were examined for their potential in papermaking, either alone or in a 50% blend with used corrugated cardboard.

The chemical composition of every solid and liquid phase generated during the MS fractionation process was determined. For the fresh green harvested hay and field-dried hay, the results revealed that the green harvest contains substantially more protein. Investigations into the chemical composition of different alfalfa fractions revealed that the leaves had a higher protein content (245) while the stems retained the majority of the carbohydrates (more than 53%). Consequently, one of the most important factors in MS biomass fractionation is the stem-to-leaf ratio.

Depending on the desired outcome, e.g., more protein content or more hemicellulose, the parameters of the pretreatment and alkaline treatment processes can be adjusted. Protein extracts, papermaking fibers, hemicelluloses, and lignin were the targets of the current investigation, and the combination of water pretreatment (E1) and soda pulping (PT3) produced the most desirable results in terms of valuable products. However, significant differences in component recovery depended on pretreatment conditions. Water-based pretreatment (E1) was most effective for protein extraction, achieving over 40% protein content in precipitated fractions. The harshest alkaline pulping (PT3: 20% NaOH, 160 °C, 60 min) yielded cellulose-rich pulp with high glucan content, while also facilitating lignin and hemicellulose recovery from black liquor. The highest lignin purity (up to 64%) was obtained from pretreated samples under severe pulping conditions. Recovery yields for hemicelluloses ranged from 10% to 20%, with xylan as the dominant polymer.

In the next biorefinery step, the pulps derived from *Medicago sativa* stems were tested for papermaking. When blended with OCC, these fibers enhanced tensile and burst strength by 35% and 70%, respectively, compared to OCC alone. These findings support the valorization of underutilized alfalfa residues and suggest a feasible biorefinery approach for protein, fiber, and polymer recovery, aligned with circular economy principles.

## Figures and Tables

**Table 1:** The experimental protocol and experiment codes for the MS biorefinery.

Pretreatments (10:1 Liquid-to-Solid Ratio, 90 min, 75 °C)				
Experiment Codes	Experimental Environments	Inputs	Output Codes	
			Solids	Liquids
E1	water	300 g o.d. MSS	E1C1	E1C1S1
			E1C2	E1C2S2
			E1C3	E1C3S3
E2	pH 12 buffer solution		-	
E3	NaOH solution (8 g/L)		-	
**Pulping experiments** (10:1 liquid-to-solid ratio)				
Experiment codes	Experimental parameters	Inputs (200 g o.d.)	Output codes *(pulps and BLs)*	
PT1	15% NaOH, 30 min, 145 °C	Raw MSS	EP1	
		MSS from E1	EP2	
		MSS from E2	EP3	
		MSS from E3	EP4	
PT2	20% NaOH, 30 min, 145 °C	Raw MSS	EP5	
		MSS from E1	EP6	
		MSS from E2	EP7	
		MSS from E3	EP8	
PT3	20% NaOH, 60 min, 160 °C	Raw MSS	EP9	
		MSS from E1	EP10	
		MSS from E2	EP11	
		MSS from E3	EP12	

**Table 2:** Chemical composition of raw alfalfa samples.

Sample	Structural Carbohydrates (%)					Other Components (%)	
	Glucan	Xylan	Galactan	Arabinan	Mannan	Protein	AIL *
MS	30.89	8.17	1.88	2.87	3.30	12.75	20.11
MSS	32.90	12.48	2.26	3.64	1.89	8.54	19.80
MSL	10.96	3.75	2.41	4.01	0.57	24.11	18.85
MS OD	16.37	5.88	3.05	3.40	1.01	22.03	14.58

* acid-insoluble lignin.

**Table 3:** Chemical composition of the solids resulting after pretreatments.

Pretreatment	Solid Yield, (%)	Structural Carbohydrates (%)					Other Components (%)	
		Glucan	Xylan	Galactan	Arabinan	Mannan	Protein	AIL
E1	75.84	32.67	7.40	1.55	2.46	1.98	5.67	22.36
E2	73.08	34.19	8.65	1.85	2.64	1.90	5.70	22.47
E3	71.63	38.60	10.24	2.32	2.26	1.58	6.67	20.67

**Table 4:** Chemical composition of the liquids resulting after pretreatments.

Pretreatment	Structural Carbohydrates (g/L)					Other Components (g/L)			% Protein	
	Glucan	Xylan	Galactan	Arabinan	Mannan	AIL	IM	OM	SM	OM
E1	0.46	1.49	0.19	0.30	0.21	0.84	3.71	12.48	19.72	25.58
E2	0.50	2.01	0.67	0.78	0.08	1.81	11.51	22.74	15.33	18.57
E3	1.86	2.22	0.47	0.76	0.11	2.63	13.17	24.39	12.33	23.61

**Table 5:** Chemical composition of the solids resulting after the water pretreatment.

Solid Fraction	Structural Carbohydrates (%)					Other Components (%)	
	Glucan	Xylan	Galactan	Arabinan	Mannan	Protein	AIL
E1C1	3.27	4.02	1.03	1.65	0.97	30.29	17.94
E2C2	2.34	3.95	1.36	1.91	0.99	43.69	15.78
E3C3	2.94	4.17	3.56	4.75	1.53	29.44	14.74

**Table 6:** Chemical composition of the solids resulting after PT1.

Experiment	Solid Yield, (%)	Structural Carbohydrates (%)					AIL (%)
		Glucan	Xylan	Galactan	Arabinan	Mannan	
EP1	49.9	49.80	11.96	0.87	0.80	0.72	22.10
EP2	59.74	52.57	11.59	0.88	0.82	0.81	22.03
EP3	59.32	51.56	11.20	0.72	0.70	0.87	21.99
EP4	55.47	54.21	11.27	0.65	0.65	0.82	20.47

**Table 7:** Chemical composition of the solids resulting after PT2.

Experiment	Solid Yield, (%)	Structural Carbohydrates (%)					AIL (%)
		Glucan	Xylan	Galactan	Arabinan	Mannan	
EP5	47.13	53.70	12.74	0.72	0.73	0.69	22.64
EP6	53.9	57.70	11.81	0.67	0.70	0.92	20.79
EP7	53.22	54.34	11.65	0.61	0.60	0.85	22.13
EP8	54.6	48.94	10.16	0.53	0.54	0.72	21.21

**Table 8:** Chemical composition of the solids resulting after PT3.

Experiment	Solid Yield, (%)	Structural Carbohydrates (%)					AIL (%)
		Glucan	Xylan	Galactan	Arabinan	Mannan	
EP9	38.43	46.58	10.17	0.43	0.57	0.54	21.11
EP10	51.15	47.55	9.24	0.34	0.52	0.37	20.52
EP11	48.45	48.42	9.77	0.35	0.44	0.47	18.86
EP12	47.41	52.31	10.15	0.38	0.46	0.79	16.91

**Table 9:** The properties of pure MSS and OCC-MSS mixed paper sheets.

Pulp Sample	OCC	EP9	EP9+OCC	EP10	EP10+OCC	EP11	EP11+OCC	EP12	EP12+OCC
Parameter									
Drainage resistance (°SR)	33	52	43	55	45.0	51	45	46	45
Tensile index (N·m/g)	28.26	38.25	32.50	47.34	34.81	44.85	39.31	45.41	37.86
Burst index (kPa∙m^2^/g)	0.90	0.70	1.06	0.90	1.14	1.22	1.34	1.42	1.54

## Data Availability

Data will be available upon request.

## Supplementary Materials

The following supporting information can be downloaded at https://www.mdpi.com/article/10.3390/polym17121709/s1: Figure S1: FTIR spectra of isolated hemicellulose samples; Figure S2: FTIR spectra of isolated purified lignin samples; Figure S3: Chromatogram of standard mixture of calibration sugars: cellobiose (12.52), glucose (14.66), xylose (15.78), galactose (16.89), arabinose (18.38), and mannose (19.9); Figure S4: Sample chromatogram of MSS hydrolysate identifying the following sugars: glucose (14.66), xylose (15.77), galactose (16.88), arabinose (18.4), and mannose (19.9). The peak at 51.5 min is furfural resulting from the acidic degradation of pentose during the hydrolysis; Table S1: Chemical composition of raw alfalfa samples (including standard deviation values); Table S2: Chemical composition of the solids resulting after pretreatments (including standard deviation values); Table S3: Chemical composition of the liquids resulting after pretreatments (including standard deviation values); Table S4: Chemical composition of the solids resulting after the water pretreatment (including standard deviation values); Table S5: Chemical composition of the solids resulting after PT1 (including standard deviation values); Table S6: Chemical composition of the solids resulting after PT2 (including standard deviation values); Table S7: Chemical composition of the solids resulting after PT3 (including standard deviation values); Table S8: Chemical composition of liquid flows resulting from treatments; Table S9: Chemical composition of separated hemicellulose samples (raw HCs); Table S10: Chemical composition of separated lignin samples (raw lignin); Table S11: The properties of pure MSS and OCC-MSS mixed paper sheets (including standard deviation values).
