# Supplementary material for: Medicago Sativa Stems—A Multi-Output Integrated Biorefinery Approach
Source: Polymers (Basel). 2025 Jun 19;17(12):1709. doi: 10.3390/polym17121709 (PMC12196887; doi:10.3390/polym17121709)
Supplement: Supplementary file 1 [file polymers-17-01709-s001.zip › polymers-3699861-supplementary.pdf]

Suplimentary material

Medicago sativa stems – a multi-output integrated biorefinery approach

Table S1 (Table 1) Chemical composition of raw alfalfa samples (including standard deviation values)

**Sample type**	**Structural carbohydrates (%)**					**Other components (%)**	
	Glucan *	Xylan	Galactan	Arabinan	Mannan	Protein	AIL
**MS**	**30.89**	**8.17**	**1.88**	**2.87**	**3.30**	**12.75**	**20.11**
	0.11	*0.76*	*0.01*	*0.04*	*0.03*	*0.85*	*0.88*
**MSS**	**32.90**	**12.48**	**2.26**	**3.64**	**1.89**	**8.54**	**19.80**
	0.89	*0.35*	*0.04*	*0.48*	*0.15*	*0.55*	*0.50*
**MSL**	**10.96**	**3.75**	**2.41**	**4.01**	**0.57**	**24.11**	**18.85**
	0.17	*0.07*	*0.09*	*0.25*	*0.40*	*1.91*	*0.02*
**MS OD**	**16.37**	**5.88**	**3.05**	**3.40**	**1.01**	**22.03**	**14.58**
	0.44	*0.28*	*0.23*	*0.23*	*0.41*	***1.1***	*0.29*

Table S2 (Table 2) Chemical composition of the solids resulted after pretreatments (including standard deviation values)

**Sample type**	**Solid yield (%)**	**Structural carbohydrates (%)**					**Other components (%)**	
		Glucan *	Xylan	Galactan	Arabinan	Mannan	Protein	AIL
E1	**75.84**	**32.67**	**7.40**	**1.55**	**2.46**	**1.98**	**5.67**	**22.36**
	2.10	*0.67*	*0.19*	*0.05*	*0.09*	*0.10*	*0.55*	*0.26*
E2	**73.08**	**34.19**	**8.65**	**1.85**	**2.64**	**1.90**	**5.70**	**22.47**
	1.22	*1.38*	*0.40*	*0.14*	*0.26*	*0.51*	*0.33*	*0.93*
E3	**71.63**	**38.60**	**10.24**	**2.32**	**2.26**	**1.58**	**6.67**	**20.67**
	1.57	*1.50*	*0.25*	*0.13*	*0.41*	*0.33*	*0.61*	*0.37*

Table S3 (Table 3). Chemical composition of the liquids resulted after pretreatments (including standard deviation values)

**Experiment**	**Structural carbohydrates (g/L)**					**Other components (g/L)**			**% protein in**	
Liquid phase	Glucan *	Xylan	Galactan	Arabinan	Mannan	AIL	IM	OM	**SM**	**OM**
E1	**0.46**	**1.49**	**0.19**	**0.30**	**0.21**	**0.84**	**3.71**	**12.48**	**19.72**	**25.58**
	0.04	*0.04*	*0.01*	*0.03*	*0.01*	*0.1*	*0.08*	*0.07*	*1.12*	
E2	**0.50**	**2.01**	**0.67**	**0.78**	**0.08**	**1.81**	**11.51**	**22.74**	**15.33**	**18.57**
	0.17	*0.04*	*0.04*	*0.01*	*0.00*	*0.05*	*0.08*	*0.24*	*0.91*	
E3	**1.86**	**2.22**	**0.47**	**0.76**	**0.11**	**2.63**	**13.17**	**24.39**	**12.33**	**23.61**
	0.04	*0.13*	*0.10*	*0.04*	*0.01*	*0.12*	*0.27*	*0.48*	*1.01*	

Table S4 (Table 4) Chemical composition of the solids resulted after the water pretreatment (including standard deviation values)

Sample typw	Carbohydrates (%)					Other components (%)	
	Glucan	Xylan	Galactan	Arabinan	Mannan	Protein	AIL
E1C1	**3.27**	**4.02**	**1.03**	**1.65**	**0.97**	**30.29**	**17.94**
	0.27	*0.02*	*0.04*	*0.00*	*0.45*	*1.14*	*2.24*
E1C2	**2.34**	**3.95**	**1.36**	**1.91**	**0.99**	**43.69**	**15.78**
	0.11	*0.10*	*0.10*	*0.04*	*0.39*	*2.01*	*1.46*
E1C3	**2.94**	**4.17**	**3.56**	**4.75**	**1.53**	**29.44**	**14.74**
	0.31	*0.42*	*0.47*	*0.06*	*0.10*	*0.97*	*0.31*

Table S5 (Table 5). Chemical composition of the solids resulted after PT1 (including standard deviation values)

Experiment	Solid yield (%)	Structural carbohydrates (%)					AIL (%)
		Glucan	Xylan	Galactan	Arabinan	Mannan	
**EP1**	**49.9**	**49.80**	**11.96**	**0.87**	**0.80**	**0.72**	**22.10**
	2.21	*1.07*	*0.37*	*0.03*	*0.03*	*0.07*	*0.71*
**EP2**	**59.74**	**52.57**	**11.59**	**0.88**	**0.82**	**0.81**	**22.03**
	1.87	*1.66*	*0.01*	*0.03*	*0.04*	*0.05*	*0.48*
**EP3**	**59.32**	**51.56**	**11.20**	**0.72**	**0.70**	**0.87**	**21.99**
	2.32	*1.75*	*0.34*	*0.04*	*0.02*	*0.00*	*0.98*
**EP4**	**55.47**	**54.21**	**11.27**	**0.65**	**0.65**	**0.82**	**20.47**
	3.11	*0.88*	*0.31*	*0.02*	*0.01*	*0.01*	*0.59*

Table S6 (Table 6). Chemical composition of the solids resulted after PT2 (including standard deviation values)

Experiment	Solid yield (%)	Structural carbohydrates (%)					AIL (%)
		Glucan*	Xylan	Galactan	Arabinan	Mannan	
**EP5**	**47.13**	**53.70**	**12.74**	**0.72**	**0.73**	**0.69**	**22.64**
	2.21	*0.48*	*0.29*	*0.05*	*0.05*	*0.09*	*0.40*
**EP6**	**53.9**	**57.70**	**11.81**	**0.67**	**0.70**	**0.92**	**20.79**
	1.15	*0.97*	*0.02*	*0.03*	*0.01*	*0.04*	*0.83*
**EP7**	**53.22**	**54.34**	**11.65**	**0.61**	**0.60**	**0.85**	**22.13**
	0.98	*1.98*	*0.16*	*0.00*	*0.01*	*0.00*	*0.51*
**EP8**	**54.6**	**48.94**	**10.16**	**0.53**	**0.54**	**0.72**	**21.21**
	1.02	*0.28*	*0.39*	*0.02*	*0.02*	*0.04*	*0.18*

Table S7 (Table 7) Chemical composition of the solids resulted after PT3 (including standard deviation values)

Experiment	Solid yield (%)	Structural carbohydrates (%)					AIL (%)
		Glucan*	Xylan	Galactan	Arabinan	Mannan	
**EP9**	**38.43**	**46.58**	**10.17**	**0.43**	**0.57**	**0.54**	**21.11**
	1.25	0.00	0.11	0.00	0.01	0.03	0.53
**EP10**	**51.15**	**47.55**	**9.24**	**0.34**	**0.52**	**0.37**	**20.52**
	2.35	*1.01*	*0.26*	*0.02*	*0.02*	*0.01*	*0.26*
**EP11**	**48.45**	**48.42**	**9.77**	**0.35**	**0.44**	**0.47**	**18.86**
	3.01	*3.49*	*0.23*	*0.01*	*0.01*	*0.01*	*0.63*
**EP12**	**47.41**	**52.31**	**10.15**	**0.38**	**0.46**	**0.79**	**16.91**
	2.34	*0.74*	*0.18*	*0.13*	*0.03*	*0.09*	0.73

Table S8 Chemical composition of liquid flows resulted from treatments

**Experiment**	**Structural carbohydrates (g/L)**				**Other components (g/L)**			
	Glucan *	Xylan	Galactan	Arabinan	AIL	ASL	IM	OM
EP1	**1.57**	**3.52**	**1.72**	**2.82**	**5.47**	**1.76**	**21.88**	**47.43**
	0.07	0.16	0.12	0.13	0.11	0.09	0.33	0.59
EP2	**0.65**	**1.94**	**1.83**	**2.92**	**6.73**	**1.51**	**20.31**	**36.50**
	0.08	0.15	0.02	0.11	0.21	0.24	1.07	1.24
EP3	**0.85**	**2.28**	**1.78**	**3.06**	**6.19**	**1.06**	**21.42**	**37.91**
	0.01	0.15	0.21	0.24	0.09	0.03	1.1	08.8
EP4	**0.91**	**2.77**	**1.66**	**3.17**	**6.52**	**1.09**	**23.95**	**33.71**
	0.07	0.11	0.21	0.29	0.23	0.06	1.07	1.78
EP5	**1.95**	**4.28**	**2.27**	**3.21**	**5.88**	**1.51**	**26.11**	**54.05**
	0.13	0.02	0.11	0.08	0.17	0.02	1.64	0.86
EP6	**0.89**	**2.61**	**1.94**	**3.39**	**7.26**	**1.25**	**23.00**	**42.56**
	0.18	0.14	0.21	0.57	0.28	0.02	1.21	1.39
EP7	**3.04**	**6.30**	**4.02**	**4.04**	**7.01**	**1.21**	**28.36**	**39.28**
	0.15	0.03	0.74	0.38	0.22	0.01	1.41	0.37
EP8	**3.47**	**7.36**	**4.02**	**4.71**	**5.98**	**1.06**	**30.98**	**33.80**
	0.03	0.31	0.10	0.18	0.14	0.04	0.15	0.11
EP9	**1.54**	**4.14**	**1.32**	**2.98**	**7.15**	**1.93**	**32.22**	**58.66**
	0.22	0.61	0.25	0.42	0.32	0.15	1.14	0.43
EP10	**0.59**	**2.70**	**1.18**	**2.76**	**7.52**	**1.39**	**25.97**	**42.82**
	0.07	0.02	0.16	0.09	0.18	0.04	0.74	0.67
EP11	**0.46**	**3.22**	**1.11**	**2.16**	**7.6**	**1.11**	**26.90**	**36.28**
	0.08	0.05	0.05	0.11	0.15	0.12	0.23	1.68
EP12	**0.41**	**3.90**	**0.96**	**2.08**	**8.53**	**1.24**	**30.71**	**33.24**
	0.01	0.04	0.06	0.03	0.22	0.06	3.69	1.10

Table S9. Chemical composition of separated hemicelluloses samples (raw HC)

Experiment	Carbohydrates (%)				
	Glucan*	Xylan	Galactan	Arabinan	Manan
EP1	**4.36**	**27.27**	**9.46**	**6.71**	**0.63**
	0.29	0.66	0.10	0.09	0.44
EP2	**5.69**	**40.31**	**9.19**	**6.59**	**0.88**
	0.30	0.13	0.41	0.94	0.07
EP3	**6.57**	**41.32**	**8.11**	**6.19**	**0.99**
	0.48	0.40	0.01	0.02	0.14
EP4	**5.26**	**45.99**	**7.32**	**5.65**	**0.80**
	0.13	0.56	0.15	0.73	0.09
EP5	**6.08**	**31.53**	**6.23**	**5.42**	**1.57**
	0.37	1.53	0.21	0.46	0.10
EP6	**4.46**	**36.45**	**5.54**	**5.15**	**1.47**
	0.17	2.18	0.53	0.25	0.92
EP7	**5.34**	**33.58**	**3.80**	**4.31**	**2.58**
	0.23	0.86	0.12	0.11	0.03
EP8	**3.37**	**33.33**	**3.52**	**4.11**	**2.97**
	0.00	0.57	0.04	0.02	0.08
EP9	**6.75**	**30.44**	**4.22**	**5.30**	**0.90**
	0.79	1.67	0.43	1.08	0.17
EP10	**4.77**	**36.51**	**4.40**	**4.77**	**1.04**
	0.17	0.30	0.02	0.41	0.30
EP11	**3.50**	**33.47**	**3.01**	**3.30**	**1.15**
	0.10	0.06	0.05	0.16	0.24
EP12	**2.68**	**33.73**	**2.67**	**3.23**	**1.78**
	0.12	0.43	0.06	0.06	0.61

Table S10 Chemical composition of separated lignin samples (raw lignin)

Experiment	Lignin (%)		Carbohydrates (%)				
	AIL	ASL	Glucan*	Xylan	Galactan	Arabinan	Manan
EP1	**42.80**	**2.11**	**2.18**	**2.89**	**1.71**	**3.28**	**0.25**
	1.28	0.05	0.01	0.07	0.06	0.09	0.01
EP2	**48.25**	**1.74**	**1.45**	**2.34**	**1.83**	**3.77**	**0.61**
	0.35	0.04	0.02	0.07	0.02	0.01	0.03
EP3	**43.50**	**1.52**	**1.32**	**2.30**	**1.72**	**3.45**	**0.89**
	1.17	0.07	0.05	0.06	0.09	0.12	0.14
EP4	**39.84**	**1.42**	**1.14**	**2.69**	**1.67**	**3.23**	**1.30**
	1.52	0.10	0.06	0.02	0.02	0.24	0.18
EP5	**46.77**	**1.69**	**1.80**	**2.34**	**1.39**	**2.53**	**1.06**
	0.19	0.03	0.07	0.07	0.10	0.48	0.80
EP6	**53.32**	**1.85**	**1.62**	**2.30**	**1.47**	**2.33**	**0.66**
	0.69	0.02	0.02	0.10	0.04	0.09	0.02
EP7	**50.44**	**1.78**	**1.52**	**2.08**	**1.34**	**2.12**	**0.49**
	0.50	0.11	0.05	0.04	0.04	0.13	0.16
EP8	**51.94**	**1.56**	**1.37**	**2.75**	**1.32**	**2.15**	**0.43**
	0.82	0.04	0.10	0.05	0.01	0.09	0.04
EP9	**57.48**	**2.15**	**1.95**	**2.27**	**0.99**	**2.07**	**0.00**
	0.51	0.07	0.03	0.20	0.04	0.07	0.00
EP10	**61.71**	**1.98**	**1.32**	**2.41**	**1.14**	**2.26**	**0.00**
	1.19	0.10	0.10	0.30	0.09	0.16	0.00
EP11	**63.76**	**1.83**	**0.98**	**2.68**	**1.06**	**2.15**	**0.00**
	0.62	0.11	0.06	0.35	0.02	0.02	0.00
EP12	**63.60**	**2.06**	**0.98**	**2.68**	**1.06**	**2.15**	**0.00**
	0.18	0.10	0.06	0.35	0.02	0.02	0.00

Table S11 (Table 8) The properties of pure MSS and OCC-MSS mixed paper sheets (including standard deviation values)

Parameter/ pulp sample	OCC	EP9	EP9+OCC	EP10	EP10+OCC	EP11	EP11+OCC	EP12	EP12+OCC
**Drainage resistance (°SR)**	33	52	43	55	45.0	51	45	46	45
**Tensile index (N·m/g)**	**28.26**	**38.25**	**32.50**	**47.34**	**34.81**	**44.85**	**39.31**	**45.41**	**37.86**
	1.29	1.73	1.38	0.55	2.09	2.11	1.55	2.32	2.02
**Burst index (kPa·m^2/g)**	**0.90**	**0.70**	**1.06**	**0.90**	**1.14**	**1.22**	**1.34**	**1.42**	**1.54**
	0.02	0.80	0.06	0.04	0.09	0.15	0.04	0.08	0.30

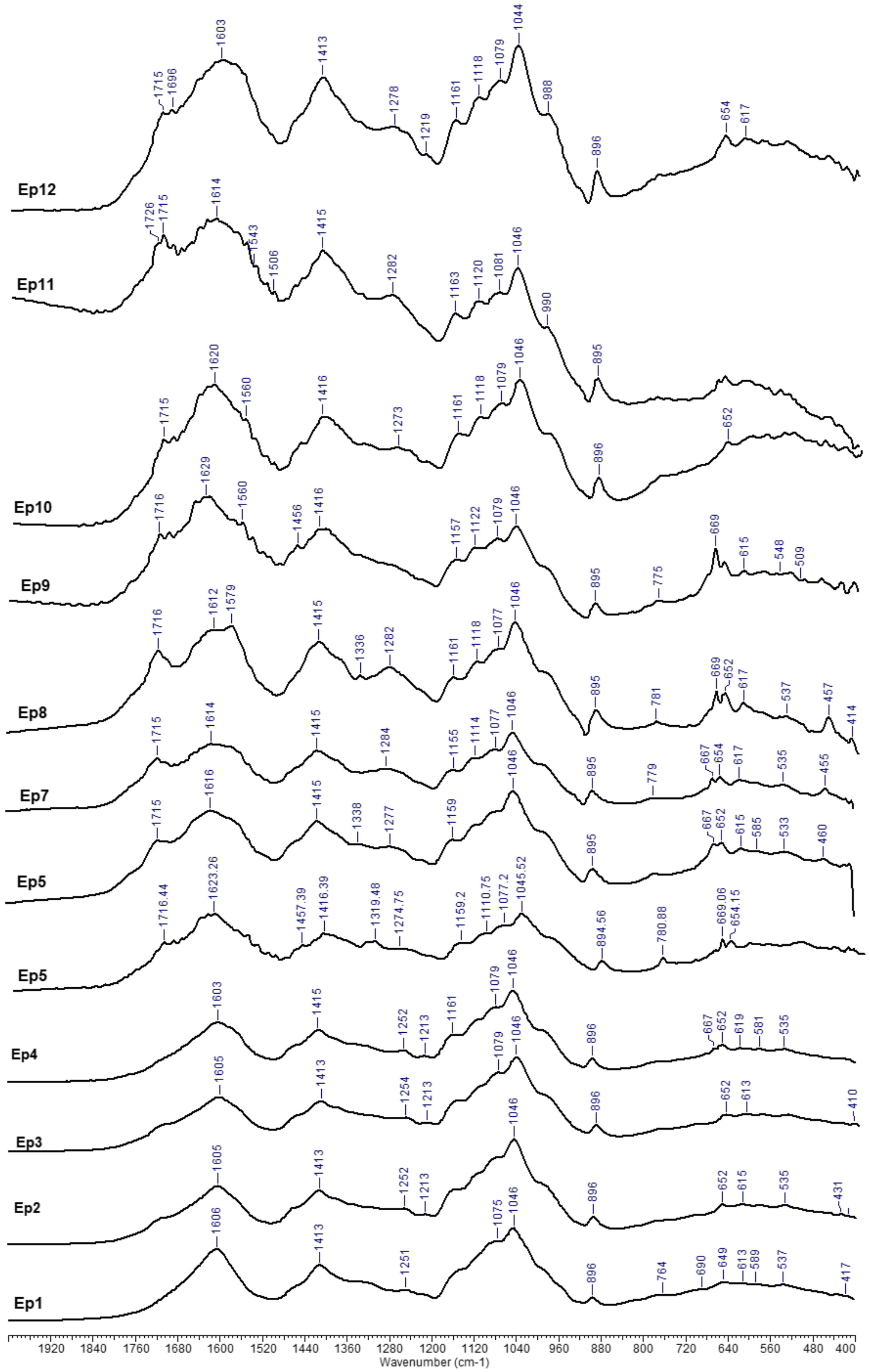

Figure S1. FTIR spectra of isolated hemicelluloses samples

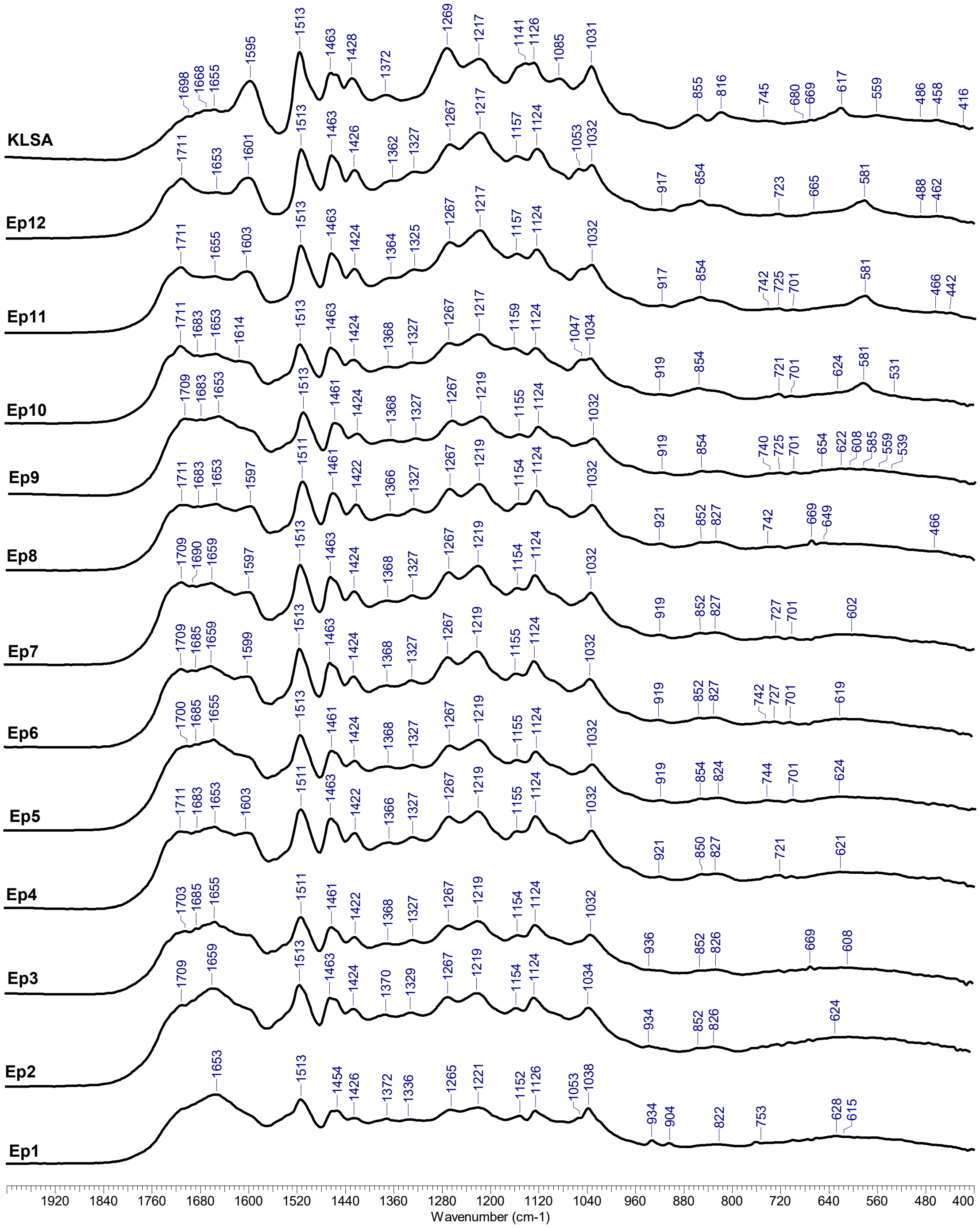

Figure S2. FTIR spectra of isolated purified lignin samples

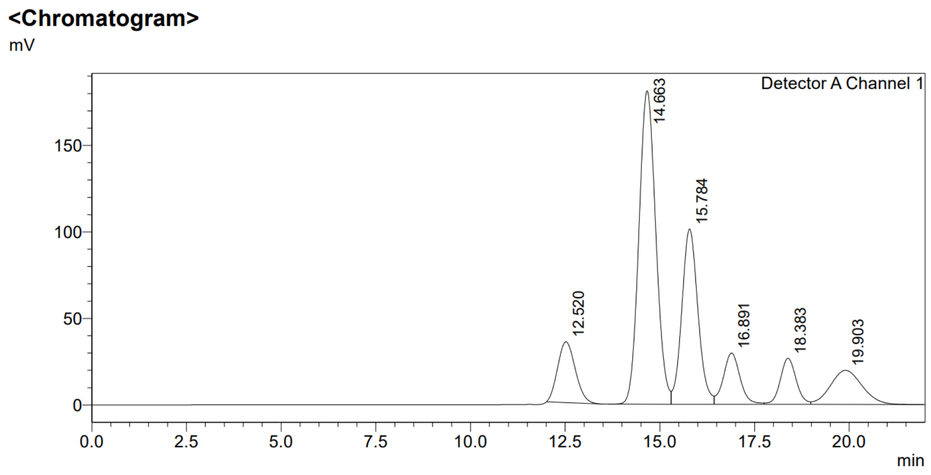

Figure S3 Chromatogram of standard mixture of calibration sugars: cellobiose (12.52), glucose (14.66), xylose (15.78), galactose (16.89), arabinose (18.38) and mannose (19.9)

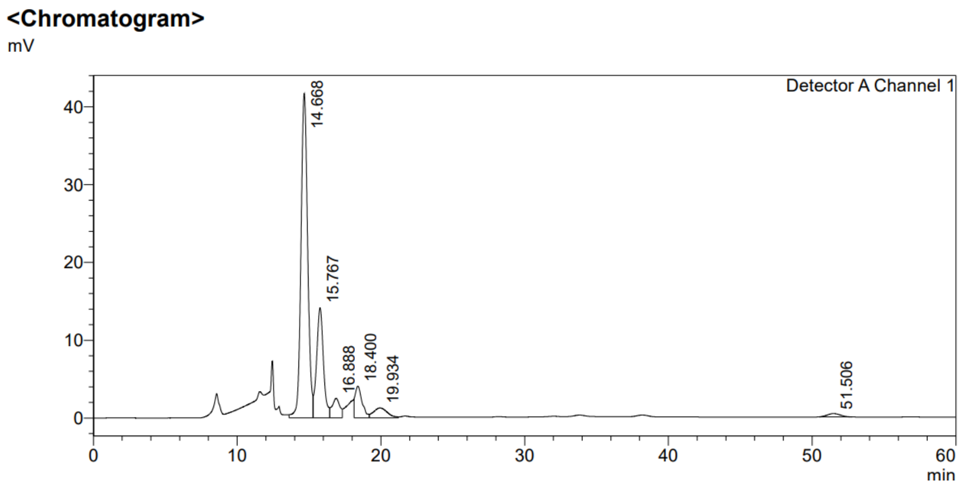

Figure S4 Sample chromatogram of MSS hydrolysate identified sugars were: glucose (14.66), xylose (15.77), galactose (16.88), arabinose (18.4) and mannose (19.9). Peak at 51.5 minutes is furfural resulted from acidic degradation of pentoses during the hydrolysis.
